# Epidemiology and related risk factors of prolonged QT interval in neonates

**DOI:** 10.12669/pjms.41.9.11825

**Published:** 2025-09

**Authors:** Yu Hong, Yongmei Wang, Qin Zhou, Min Wang, Jing Zhou

**Affiliations:** 1 Yu Hong Department of Pediatrics, The People’s Hospital of Suzhou New District, Suzhou 215000, Jiangsu, China; 2 Yongmei Wang Department of Pediatrics, The People’s Hospital of Suzhou New District, Suzhou 215000, Jiangsu, China; 3 Qin Zhou Department of Pediatrics, The People’s Hospital of Suzhou New District, Suzhou 215000, Jiangsu, China; 4 Min Wang Department of Pediatrics, The People’s Hospital of Suzhou New District, Suzhou 215000, Jiangsu, China; 5 Jing Zhou Department of Pediatrics, The People’s Hospital of Suzhou New District, Suzhou 215000, Jiangsu, China

**Keywords:** Epidemiology, Influencing factor, Neonate, Prolonged QT interval

## Abstract

**Objective:**

To investigate the epidemiology and related risk factors of prolonged QT interval (PQTI) in neonates.

**Methodology:**

This was a retrospective study. A cohort of one thousand neonates admitted to the Obstetrics and Neonatology department of The People’s Hospital of Suzhou New District from January 2021 to December 2023 was enrolled in the study. Neonates were categorized into four groups based on their birth gestational age: healthy full-term group, diseased full-term group, healthy preterm group, and diseased preterm group. The incidence of PQTI in neonates across these group was observed, compared and investigated the risk factors for PQTI. The diagnostic value of QTcHod, QTcFri, and QTcFra was assessed using Receiver Operating Characteristic (ROC) curve analysis.

**Results:**

Among the 1,000 neonates, 51(5.10%) exhibited PQTI. The incidence of PQTI was 0.98% (7/714) for Group-1, 3.64%(2/55) for Group-2, 15.05%(28/186) for Group-3, and 31.11%(14/45) for Group-4. Statistically significant differences were observed in the incidence of PQTI between the four groups(χ² = 137.637, *P* < 0.001, respectively). Statistically significant differences were found in the QTcBaz levels across the four groups(P< 0.05, respectively). The ROC curve analysis showed that the area under the curve for QTcHod, QTcFri, and QTcFra in diagnosing PQTI was 0.785, 0.741, and 0.812, with sensitivities of 78.40%, 74.60%, and 89.30%, respectively, indicating their high diagnostic value.

**Conclusion:**

PQTI is relatively uncommon in neonates. Birth gestational age, 1-minute Apgar score, 5-minute Apgar score, PROM, fetal distress, QTcHod, QTcFri, and QTcFra are significant risk factors for PQTI in neonates.

## INTRODUCTION

Long QT Syndrome (LQTS) is a potentially fatal arrhythmia that can lead to sudden cardiac death. With timely intervention, the mortality rate can be reduced to below 1%.^1^ Previous studies^2^ have shown that children with LQTS often have no structural heart abnormalities, and postmortem examinations rarely reveal any irregularities, earning it the moniker “silent killer” in neonates. Many researchers^3^-^5^ consider LQTS to be characterized by prolonged ventricular repolarization, which is reflected as a prolonged QT interval (PQTI) on the surface electrocardiogram (ECG). PQTI is a specialized clinical term used to describe the time between the start of the QRS complex and the end of the T wave on the ECG that exceeds the normal range.

As early as 20 years ago, European countries began including neonatal surface ECG screening as part of their cardiovascular screening programs, which has been beneficial for initiating early preventive treatment in LQTS-affected neonates and, in turn, reducing the incidence of sudden death in this population.^6^ In contrast, reports on PQTI in neonates remain limited in China, and most of the available literature is quite old.^7^ Therefore, there is a lack of current epidemiological data and studies on the risk factors associated with PQTI in neonates in China. This study seeks to fill this gap, providing theoretical insights for the diagnosis and management of PQTI in neonates.

## METHODOLOGY

This was a retrospective study. A total of 1,000 neonates admitted to the Obstetrics and Neonatology departments at The People’s Hospital of Suzhou New District between January 2021 to December 2023 were enrolled in the study. Factors that could potentially influence the occurrence of PQTI in neonates were collected, including sex, birth gestational age, birth weight, delivery type, 1-minute Apgar score, 5-minute Apgar score, placenta previa, placental abruption, premature rupture of membranes (PROM), fetal distress, gestational hypertension, gestational diabetes, as well as QTcHod, QTcFri, and QTcFra values.

### Ethical Approval:

The study was approved by the Institutional Ethics Committee of The People’s Hospital of Suzhou New District (No.: 2022-076; Date: December 23, 2022), and written informed consent was obtained from the parents of all participating neonates.

### Inclusion criteria:

- The diagnostic cutoff for PQTI in neonates is QTcBaz > 440 ms or > 460 ms
- QTcBaz > 440 ms being the traditional diagnostic criterion.^8^,^9^
- Parents signed informed consent forms at the hospital.

### Exclusion criteria

- Infants with congenital heart disease.
- Infants with genetic metabolic disorders.
- Infants with severe congenital organ or systemic malformations.

### Screening for PQTI in Neonates:

QTcBaz is considered the most stable and reliable parameter for neonates, capable of accurately identifying those at high risk for LQTS. Therefore, in this study, QTcBaz > 440 ms was used as the diagnostic criterion for PQTI in neonates. A 12-lead surface ECG was recorded within 48 hours of birth in a quiet environment. The QTc interval was calculated using an online QTc calculator (https://www.thecalculator.co/health/QTc-Calculator-385.html) by inputting the heart rate (bpm) and QT interval (ms). This provided the QTcBaz, QTcHod, QTcFri, and QTcFra values. If the QTcBaz was > 440 ms on the first ECG, the neonate was re-assessed with a second ECG before discharge. If the QTcBaz remained > 440 ms in the second ECG, the neonate was diagnosed with PQTI. The same method was used to calculate QTcBaz, QTcHod, QTcFri, and QTcFra.

### Grouping

The criteria for assessing diseased neonates include feeding difficulties, fever, pathological jaundice, tachypnoea, apnea, antibiotic use for more than three days, weakened primitive reflexes, grunting, and positive blood cultures.^10^ Based on disease status and birth gestational age, the neonates were divided into four groups: Group-1 (healthy full-term neonates): 714 neonates, no disease, born full-term (gestational age ≥ 37 weeks). Group-2 (diseased full-term neonates): 55 neonates, affected by disease (meeting the diagnostic criteria for diseased neonates), born full-term. Group-3 (healthy preterm neonates): 186 neonates, no disease, born preterm (gestational age < 37 weeks). Group-4 (diseased preterm neonates): 45 neonates, affected by disease, born preterm. Furthermore, neonates were classified into two subgroups based on the presence or absence of PQTI: the prolongation group and the non-prolongation group.

### Outcome Measures:

- To assess the overall incidence of PQTI in neonates, and to calculate and compare the incidence rates of PQTI across different groups.
- To compare the QTcBaz levels across the four groups.
- To identify factors associated with the occurrence of PQTI in neonates using logistic regression analysis.
- To evaluate the diagnostic value of QTcHod, QTcFri, and QTcFra in detecting PQTI in neonates through ROC curve analysis.

### Statistical analysis:

Data were processed using the SPSS 26.0 software, with a significance level set at *α* = 0.05. Measurement data were presented as mean ± standard deviation (*X̅*±*S*) and compared using t-tests. Categorical data were analyzed using the chi-square (*χ*²) test. Logistic regression was used for risk factor analysis, and ROC curves were employed to assess predictive performance.

## RESULTS

Among the 1,000 neonates, 51 were found to have PQTI, resulting in an overall incidence of 5.10% (51/1,000). In Group-1 (*n =* 714), three neonates had PQTI, corresponding to an incidence of 0.98% (7/714). In Group-2 (*n =* 55), 2 neonates had PQTI, with an incidence of 3.64% (2/55). In Group-3 (*n =* 186), 28 neonates had PQTI, yielding an incidence of 15.05% (28/186). In Group-4 (*n =* 45), 14 had PQTI, with an incidence of 31.11% (14/45). Statistically significant differences were observed in the incidence of PQTI between the four groups (*χ*² = 137.637, *P <* 0.001, respectively). Specifically, the incidence of PQTI was significantly higher in Groups-3 and 4 compared with Group-1 (*χ*² = 78.193, 142.855, *P <* 0.001, respectively). Likewise, PQTI incidence was also significantly higher in Groups-3 and 4 compared with Group-2 (*χ*² = 5.077, 13.901, *P =* 0.024, < 0.001). Additionally, Group-4 showed a significantly higher incidence than Group-3 (*χ*² = 6.280, *P =* 0.012). No significant difference was found between Group-1 and Group-2 in PQTI incidence (*χ*² = 3.114, *P =* 0.078).

The comparison of QTcBaz levels across the four groups displayed significant statistical differences (*P <* 0.05, respectively). Specifically, Groups-3 and 4 had significantly higher QTcBaz levels compared with Group-1 (*P <* 0.05, respectively). Similarly, QTcBaz levels in Groups-3 and 4 were higher than in Group-2 (*P <* 0.05, respectively). Additionally, Group-4 exhibited significantly higher QTcBaz levels than Group-3 (*P <* 0.05). No significant difference in QTcBaz levels was found between Group-1 and Group-2 (*P >* 0.05) (Table-I).

**Table-I:** Comparison of QTcBaz levels across the four groups.

Group	Case (n)	QTcBaz (ms)
Group-1	714	403.58±54.36
Group-2	55	405.35±50.34
Group-3	186	429.32±48.75^ab^
Group-4	45	451.76±43.57^abc^
F-value		21.372
P-value		<0.001

***Notes:*** aP < 0.05 compared with Group-1;

bP < 0.05 compared with Group-2;

cP < 0.05 compared with Group-3.

Compared with the non-prolongation group, the prolongation group showed no significant differences in sex, birth weight, delivery type, placental previa, gestational hypertension, or gestational diabetes (all *P >* 0.05). However, significant differences were observed between the two groups in birth gestational age, 1-minute Apgar score, 5-minute Apgar score, PROM, fetal distress, QTcHod, QTcFri, and QTcFra (*P <* 0.05, respectively) (Table-II).

**Table-II:** Univariate analysis of factors associated with PQTI in neonates.

Item	Prolongation group (n = 51)	Non-prolongation group (n = 949)	χ²/t-value	P-value
***Sex***			0.964	0.326
Male	26(50.98)	550(57.96)		
Female	25(49.02)	399(42.04)		
***Birth gestational age***			106.215	<0.001
<37 weeks	42(82.35)	189(19.92)		
≥37 weeks	9(17.65)	760(80.08)		
***Birth weight***			0.244	0.885
<2,500 g	5(9.80)	75(7.90)		
2,500–4,000 g	29(56.86)	546(57.53)		
> 4,000 g	17(33.33)	328(34.56)		
***Delivery type***			0.049	0.825
Vaginal	21(41.18)	376(39.62)		
Cesarean	30(58.82)	573(60.38)		
***1-minute Apgar score***			301.638	<0.001
8–10	1(1.96)	828(87.25)		
4–7	45(88.24)	121(12.75)		
≤3	5(9.80)	0(0.00)		
***5-minute Apgar score***			287.403	<0.001
8–10	2(3.92)	825(86.93)		
4–7	43(84.31)	123(12.96)		
≤3	6(11.76)	1(0.11)		
***Placenta previa***			0.970	0.325
Yes	6(11.76)	75(7.90)		
No	45(88.24)	874(92.10)		
***PROM***			92.740	<0.001
Yes	20(39.22)	46(4.85)		
No	31(60.78)	903(95.15)		
***Fetal distress***			12.016	0.001
Yes	29(56.86)	315(33.19)		
No	22(43.14)	634(66.81)		
***Gestational hypertension***			1.231	0.267
Yes	19(37.25)	284(29.93)		
No	32(62.75)	665(70.07)		
***Gestational diabetes***			2.385	0.123
Yes	20(39.22)	276(29.08)		
No	31(60.78)	673(70.92)		
QTcHod (ms)	457.43±52.34	405.32±42.15	8.462	<0.001
QTcFri (ms)	452.18±51.63	404.52±39.41	8.266	<0.001
QTcFra (ms)	449.84±42.87	401.13±36.42	9.216	<0.001

In this study, logistic regression was used to analyze the risk factors associated with PQTI in neonates, using the occurrence of PQTI as the dependent variable (Y), and birth gestational age, 1-minute Apgar score, five minutes Apgar score, PROM, fetal distress, QTcHod, QTcFri, and QTcFra as independent variables (X). The results are summarized in Table-III. The logistic regression analysis revealed the following significant risk factors for PQTI in neonates: birth gestational age, one minute Apgar score, five minute Apgar score, PROM, fetal distress, QTcHod, QTcFri, and QTcFra (*P <* 0.05, respectively) (Table-IV).

**Table-III:** Variable assignments for logistic regression analysis.

Variable Name	Variable	Assignment
Birth gestational age	X_1_	≥37 weeks = 0, <37 weeks = 1
1-minute Apgar score	X_2_	8–10 points = 0, 4–7 points = 1, ≤3 points = 2
5-minute Apgar score	X_3_	8–10 points = 0, 4–7 points = 1, ≤3 points = 2
PROM	X_4_	No = 0, Yes = 1
Fetal distress	X_5_	No = 0, Yes = 1
QTcHod	X_6_	Continuous variable
QTcFri	X_7_	Continuous variable
QTcFra	X_8_	Continuous variable

**Table-IV:** Multivariate analysis of risk factors for PQTI in neonates.

Variable	Regression coefficient	Standard error	Wald χ^2^ value	OR value	95% CI		P-value
					Minimum value	Maximum value	
Birth gestational age	1.234	0.316	15.250	3.435	1.783	5.087	<0.001
1-minute Apgar score	1.768	0.334	28.020	5.859	3.356	8.362	<0.001
5-minute Apgar score	1.587	0.324	23.992	4.889	2.724	7.054	<0.001
PROM	1.106	0.307	12.979	3.022	1.629	4.415	<0.001
Fetal distress	0.879	0.296	8.818	2.408	1.414	3.403	<0.001
QTcHod	0.764	0.275	7.718	2.147	1.269	3.025	0.002
QTcFri	0.683	0.268	6.495	1.980	1.207	2.753	0.009
QTcFra	0.802	0.284	7.975	2.230	1.344	3.116	<0.001

**Fig.1:**
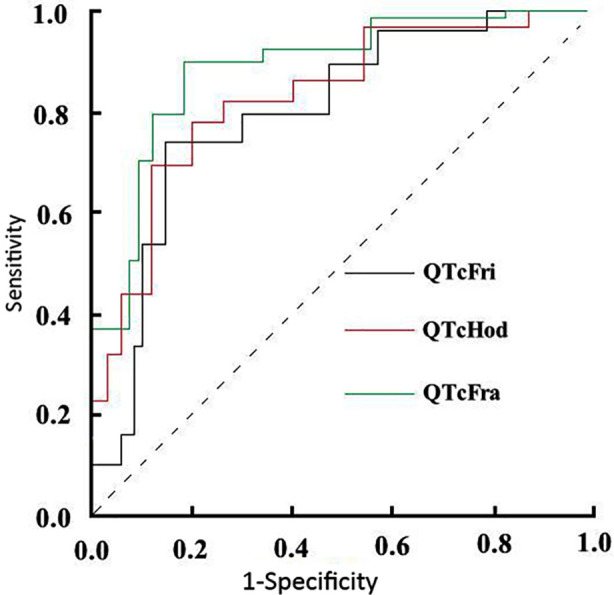
ROC curves of QTcHod, QTcFri, and QTcFra in diagnosing PQTI in neonates.

The ROC curve analysis showed that the area under the curve (AUC) values for QTcHod, QTcFri, and QTcFra in diagnosing PQTI in neonates were 0.785, 0.741, and 0.812, with sensitivities of 78.40%, 74.60%, and 89.30%, respectively. These results suggest that QTcHod, QTcFri, and QTcFra all have relatively high diagnostic value for detecting PQTI in neonates (Fig.1 and Table-V).

**Table-V:** Diagnostic value of QTcHod, QTcFri, and QTcFra for PQTI in neonates.

Parameter	Optimal Cutoff Point	Sensitivity (%)	Specificity (%)	P-value	AUC	95% CI
QTcHod	445 ms	78.40	79.30	<0.001	0.785	0.635–0.869
QTcFri	436 ms	74.60	80.50	<0.001	0.741	0.601–0.854
QTcFra	442 ms	89.30	75.20	<0.001	0.812	0.678–0.949

## DISCUSSION

In this study, we noted that 51 (5.10%) of the 1,000 study subjects exhibited PQTI, a finding consistent with several international studies, which suggests that the overall risk of PQTI in neonates is relatively low. Moreover, the neonates were divided into four groups by birth gestational age and health status, and the incidence of PQTI was examined in each group. The results revealed that the incidence of PQTI was 0.98% (7/714) in Group-1, 3.64% (2/55) in Group-2, 15.05% (28/186) in Group-3, and 31.11% (14/45) in Group-4. The incidence of PQTI differed significantly across the four groups. Neonates in Group-3 and Group-4 had higher rates of PQTI. These findings are consistent with the study by Shabestari et al.^11^, indicating that the occurrence of PQTI in neonates is closely associated with birth gestational age and the presence of comorbidities.

However, further clinical validation is necessary. LQTS is an autosomal dominant ion channel disorder, and it is responsible for approximately 20% of cases of sudden infant death syndrome (SIDS)^12^,^13^, a condition occurring typically during sleep unexpectedly. Even autopsy fails to identify a clear cause of SIDS, making it the leading cause of death among infants under one year of age.^14^ Research has indicated that PQTI in neonates during the first week of life is predictive of SIDS, which could potentially serve as an early screening tool for identifying newborns at high risk of SIDS.^15^ This highlights the critical role of surface ECG in detecting PQTI in neonates shortly after birth.

In our study, comparisons of QTcBaz levels among the four groups revealed statistically significant differences. Neonates in Group-3 and Group-4 had higher QTcBaz levels, which indirectly suggests that prematurity and comorbidities may elevate the risk of PQTI in neonates. To further investigate the risk factors for PQTI, the neonates were divided into two groups for univariate analysis: a group with PQTI (prolongation group) and the other without (non-prolongation group). The results revealed significant differences between the two groups in birth gestational age, one minute and five minutes Apgar scores, PROM, fetal distress, and QTcHod, QTcFri, and QTcFra levels. This indicates that the occurrence of PQTI in neonates is associated with numerous factors. The logistic regression analysis indicated that birth gestational age, 1-minute and five minutes Apgar scores, PROM, fetal distress, QTcHod, QTcFri, and QTcFra are all significant risk factors for PQTI in neonates. This suggested that the presence of more of these factors increases the risk of PQTI. The explanations for these associations are as follows: (1) Birth Gestational Age: Compared with full-term neonates, preterm neonates have less developed organs, which increases the risk of abnormalities in respiratory, organ, and circulatory systems.^16^ Consequently, preterm neonates are at a higher risk for PQTI. (2) one minute and five minutes Apgar Scores: Lower Apgar scores at one and five minutes suggest hypoxia in neonates, which can result from underdeveloped lungs, respiratory failure, or heart disease. Hypoxia may lead to myocardial ischemia and increase the risk of PQTI.^17^ (3) PROM and Fetal Distress: PROM and fetal distress can lead to fetal hypoxia, causing respiratory difficulties and affecting heart rate, thereby increasing the risk of PQTI.^17^-^19^

QTcHod, QTcFri, and QTcFra are heart rate-corrected QT interval calculation parameters. Clinically, QT intervals are often corrected for heart rate, with common formulas including QTcBaz, QTcHod, QTcFri, and QTcFra. Currently, QTcBaz > 440 ms is widely accepted as the diagnostic threshold for PQTI in neonates. However, the role of QTcHod, QTcFri, and QTcFra in evaluating PQTI in neonates still remains unclear. The results of this study indicate a strong association between these parameters and the occurrence of PQTI in neonates. The ROC curve analysis revealed that the AUC for QTcHod, QTcFri, and QTcFra in diagnosing PQTI in neonates was 0.785, 0.741, and 0.812, with sensitivities of 78.40%, 74.60%, and 89.30%, respectively. These results suggest that QTcHod, QTcFri, and QTcFra have significant diagnostic value for identifying PQTI in neonates. This further confirms the high clinical value of these parameters for PQTI assessment and supports their potential for broader clinical application.

### Limitations:

However, the limitation of this study is a small number of patients and it was a retrospective descriptive study, with limited clinical data available and limited persuasive conclusions. In view of this, further improvements will be made in future research to make more scientific research results.

## CONCLUSIONS

The incidence of PQTI in neonates is relatively low. Risk factors for PQTI in neonates include birth gestational age, one minute and five minutes Apgar scores, PROM, fetal distress, as well as QTcHod, QTcFri, and QTcFra levels. Additionally, QTcHod, QTcFri, and QTcFra demonstrate high diagnostic value for detecting PQTI in neonates.

### Authors’ Contributions:

**YH** and **YW:** Carried out the studies, participated in collecting data, and drafted the manuscript, and are responsible and accountable for the accuracy or integrity of the work. Critical review. **QZ** and **MW:** Literature search**,** Drafted the article and revised it critically for important intellectual content. **JZ:** Prepared the manuscript and did the statistical analysis. All authors read and approved the final manuscript.
